# Label-based routing for a family of small-world Farey graphs

**DOI:** 10.1038/srep25621

**Published:** 2016-05-11

**Authors:** Yinhu Zhai, Yinhe Wang

**Affiliations:** 1 School of Information Engineering, Guangdong University of Technology, Guangzhou, 510006, China; 2 School of Automation, Guangdong University of Technology, Guangzhou, 510006, China

## Abstract

We introduce an informative labelling method for vertices in a family of Farey graphs, and deduce a routing algorithm on all the shortest paths between any two vertices in Farey graphs. The label of a vertex is composed of the precise locating position in graphs and the exact time linking to graphs. All the shortest paths routing between any pair of vertices, which number is exactly the product of two Fibonacci numbers, are determined only by their labels, and the time complexity of the algorithm is O(n). It is the first algorithm to figure out all the shortest paths between any pair of vertices in a kind of deterministic graphs. For Farey networks, the existence of an efficient routing protocol is of interest to design practical communication algorithms in relation to dynamical processes (including synchronization and structural controllability) and also to understand the underlying mechanisms that have shaped their particular structure.

Deterministic models have unique advantages in improving our comprehension about some important physical mechanisms in complex networks. Especially, in comparison with the empirical and random models, the solutions of deterministic graphs can be obtained by rigorous derivation, and the computation is ended by a small amount of calculation. A lot of deterministic models have been created imaginatively and studied carefully, which are inspired by simple recursive operation^1^,^2^ or the techniques of plane filling^3^ or generating processes of fractal^4^ or even the relationship between natural numbers^5^. The models always have important properties similar to random models, such as scale-free and small-world and high clustered, thus it could be used to imitating empirical networks appropriately. Recently, on the basis of the classical Farey sequences, Zhang *et al*. introduced Farey graphs in ref. 6. Farey graphs are simultaneously minimally 3-colorable, uniquely Hamiltonian, and maximally outer-planar and perfect^6^,^7^. The merger of three Farey graphs coincide with the network created by edge iterations^8^, or evolving networks with geographical attachment preference^9^, or the general geometric growth model for pseudofractal scale-free web with parameter *q* = 2 and *m* = 1^10^. The combination of six Farey graphs generated the networks with multidimensional growth^11^. The graphs in refs 6, 7, 8, 9, 10, 11 are named as Farey graphs here for they are all composed on the basis of Farey graphs. Many properties of Farey graphs are comparable to those of networks associated with technological and biological systems with a high clustering and small-world, like some electronic circuits and protein networks.

Networks are very often studied considering branch of discrete mathematics known as graph theory. One active subject in graph theory is graph labelling. This is not only due to its theoretical importance but also because of the wide range of applications in many fields, such as crystallography, coding theory, circuit design and communication design^12^. Finding shortest paths in networks is a well-studied and important problem with also many applications. The all-pairs shortest paths (APSP) problem is unquestionably one of the most well-known problems in algorithm design, frequently studied in textbooks; yet, the complexity of the problem has remained open to this day. For arbitrary dense (directed and undirected) real-weighted graphs, the classical algorithms run in sub-cubic time *O*(|*V*|^3−*δ*^), where *δ* > 0^13^. The *K* shortest path routing algorithm is an extension algorithm of the shortest path routing algorithm in a given network^14^. The algorithm not only finds the shortest path, but also *K* − 1 other paths in order of increasing cost. *K* is the number of shortest paths to find. If the shortest path is not unique and *K* is small enough, the *K* shortest path routing algorithm in Farey graphs will shrink to finding out all the shortest paths.

The labelling and the routing in several deterministic models, on the basis of the relationship between vertices labels and the shortest paths, have been pioneered by Comellas and Zhang^12^,^15^,^16^. The graphs of which have some important properties similarly as empirical networks, for example, the expanded Apollonian networks are simultaneously scale-free, small-world, Euclidean, space filling, and with matching graphs^12^. Only by their labels, one of the shortest paths between any pair of vertices is determined just by simple rules and few computations^12^,^15^,^16^.

However, the research on the label-based routing for a family of Farey graphs is still lacking. We are inspired by refs 12, 15 and 16, and give a vertex labelling for Farey graphs in this paper, so that queries for all the shortest paths between any two vertices can be efficiently answered thanks to it. It is the first algorithm that calculates all the shortest paths routings of a kind of deterministic graphs. Our labelling may be useful in aspects such as network optimization, information dissemination and so on, which are directly related to the problem of finding shortest paths between all pairs of vertices of the network, and which may be of interest to understand the underlying mechanisms that have shaped their particular structure.

## Results

### Generation of Farey graphs

The generation of Farey graphs is shown as below.

**Figure 1:**
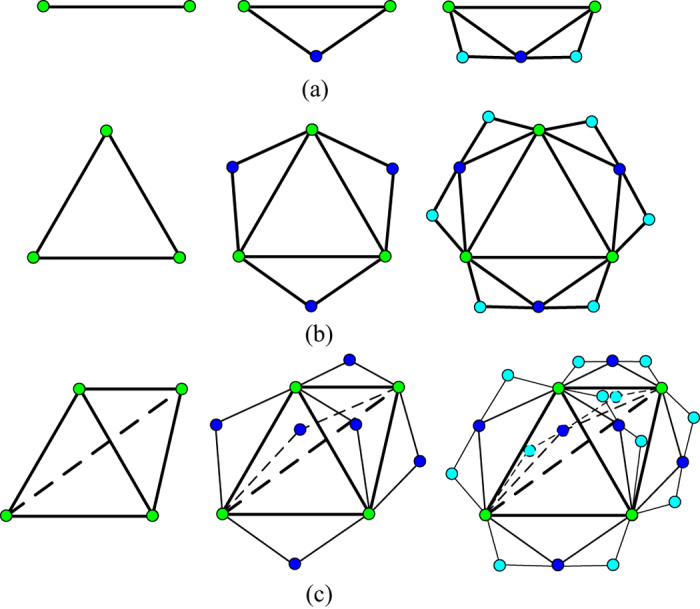
Farey graphs produced at iterations t = 0, 1 and 2. (**a**) Farey graphs *F*(*t*). (**b**) Farey graphs *N*(*t*) for *q* = 2 and *m* = 1. (**c**) Farey graphs *DMG*(*t*).

**Definition 1.** The Farey graphs *F*(*t*) = (*V*(*t*), *E*(*t*)), *t* ≥ 0, with vertex set *V*(*t*) and edge set *E*(*t*) are constructed as follows^6^:

- For *t* = 0, *F*(0) has two initial vertices and an edge joining them.
- For *t* ≥ 1, *F*(*t*) is obtained from *F*(*t* − 1) by adding to every edge introduced at step *t* − 1 a new vertex adjacent to the end vertices of this edge (see Fig. 1a).

**Definition 2.** The Farey graphs *N*(*t*) are generated as follow:

- For *t* = 0, *N*(0) has three initial vertices and an edge joining any two vertices.
- For *t* ≥ 1, by linking a new vertex to the two vertices of every edge adding at step *t* − 1, *N*(*t*) is deduced from *N*(*t* − 1) (see Fig. 1b).

**Remark 1.** The model is starting from three edges of a triangle; it is exactly the graphs created by edge iterations *N*(*t*)^8^, or evolving networks with geographical attachment preference^9^, or a general geometric growth model for pseudofractal scale-free web with parameter *q* = 2 and *m* = 1^10^.

**Definition 3.** When the model is starting from six edges of a regular tetrahedron, the Farey graphs *DMG*(*t*) are obtained by the same construction mechanism (see Fig. 1c)^11^.

Farey graphs are obviously generated by starting from an edge with two vertices. From the construction method, it is easy to get the number of vertices adding to graphs at step *t* which is Δ*n*_*t*_ = 2^*t*−1^, so that the order and size of Farey graphs are |*V*(*t*)| = 2^*t*^ + 1 and |*E*(*t*)| = 2^*t*+1^ + 1, respectively. The cumulative degree distribution of *F*(*t*) follows an exponential distribution with 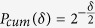 and the degree correlations *k*_*nn*_(*δ*) is approximately a linear function of *δ*, which suggests that Farey graphs are assortative. The average paths length, which is *μ*(*F*(*t*)) = [(6*t* − 5) × 2^2*t*^ + (6*t* + 17) × 2^*t*^ + (−1)^*t*^ + 5]/[9 × 2^*t*^ × (2^*t*^ + 1)], is in direct proportion to the logarithmic scale of the network’s order, so that Farey graphs is with a characteristic of small-world.

When *t* ≥ 2, any new vertex adding to *F*(*t*) at step *t* will link to two vertices: a mother vertex and a father vertex. The mother joins in graph at step *t* − 1, while the father adds to graphs at step *t* − 2 or earlier. The two vertices with the same mother are called brothers.

**Figure 2:**
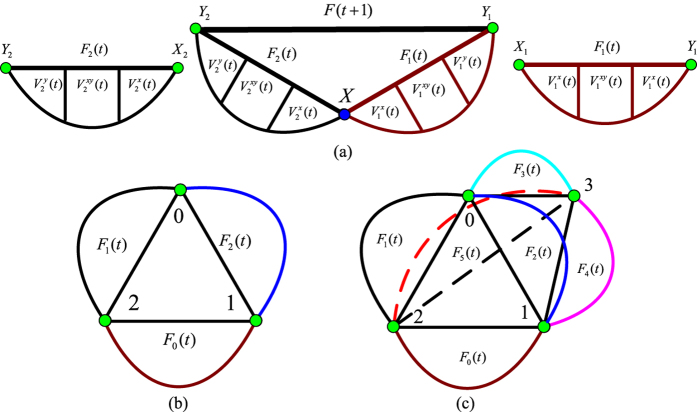
Schematic illustration of the recursive construction of the Farey graphs. (**a**) *F*(*t* + 1) is constructed of two copies of *F*(*t*). (**b**) *N*(*t*) is consisted of three copies of *F*(*t*). (**c**) *DMG*(*t*) is the merger of six copies of *F*(*t*).

By marking two initial vertices in *F*(*t*) with *X* and *Y*, all the vertices in *F*(*t*) can be divided into three groups by their distances to *X* and *Y*. Noticing that, the difference of the two distances is 0 or 1, for *X* and *Y* are neighbors. The vertices in the set *V*^*x*^(*t*) (including *X*) have shorter distances from them to *X* than to *Y*, while the vertices in *V*^*y*^(*t*) (including *Y*) have shorter distances to *Y* than to *X*, if the distances are equal, vertices are all in *V*^*xy*^(*t*). That is to say, 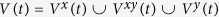. From definition 1 and the classification of vertices above, if two copies of *F*(*t*) are named as *F*_1_(*t*) and *F*_2_(*t*), *F*(*t* + 1) is generated just by merging *X*_1_ and *X*_2_ into *X* and linking *Y*_1_ and *Y*_2_ directly. The schematic diagram is shown as Fig. 2a.

The Farey graph *N*(*t*) is the combination of three Farey graphs marking with *F*_*g*_(*t*) from Fig. 1(b), where *g* ∈ {0, 1, 2}, which is described as Fig. 2b.

For the networks with multidimensional growth are constructed from six edges of a regular tetrahedron, obviously *DMG*(*t*) is the consolidation of six Farey graphs *F*_*q*_(*t*), where *q* ∈ {0, 1, 2, 3, 4, 5}, and which is shown in Fig. 2c,

### Labelling of Farey graphs

We here describe a method to label the vertices of *F*(*t*), such that all routing by shortest paths between any two vertices of *F*(*t*) are deduced from their labels.

**Definition 4.** The labelling of any vertex *v* in *F*(*t*) is performed according to the following rules:

- Label the two initial vertices with labels 0.0 and 0.1.
- At any step *t* ≥ 1, when 2^*t*−1^ new vertices are added and joined to *F*(*t* − 1), label them with labels from *t*.1 to *t*.2^*t*−1^ in clockwise direction.

**Definition 5.** The vertices in *N*(*t*) are labelled according to the following rules:

- The three initial vertices are labelled with 0.0, 0.1 and 0.2.
- Label the 3 × 2^*t*−1^ new vertices, which are joined to *N*(*t* − 1) at step *t*, with labels from *g*.*t*.1 to *g*.*t*.2^*t*−1^ in clockwise direction, where *g* ∈ {0, 1, 2} indicates which group the new vertex belongs to.

**Definition 6.** Label the vertices in *DMG*(*t*) as below:

- Label the four initial vertices with 0.0, 0.1, 0.2 and 0.3.
- For the 6 × 2^*t*−1^ new vertices which are joined to *DMG*(*t* − 1) at step *t*, the labels of them are from *g*.*t*.1 to *g*.*t*.2^*t*−1^ in a certain order, where the group indicator is *g* ∈ {0, 1, 2, 3, 4, 5}.

**Figure 3:**
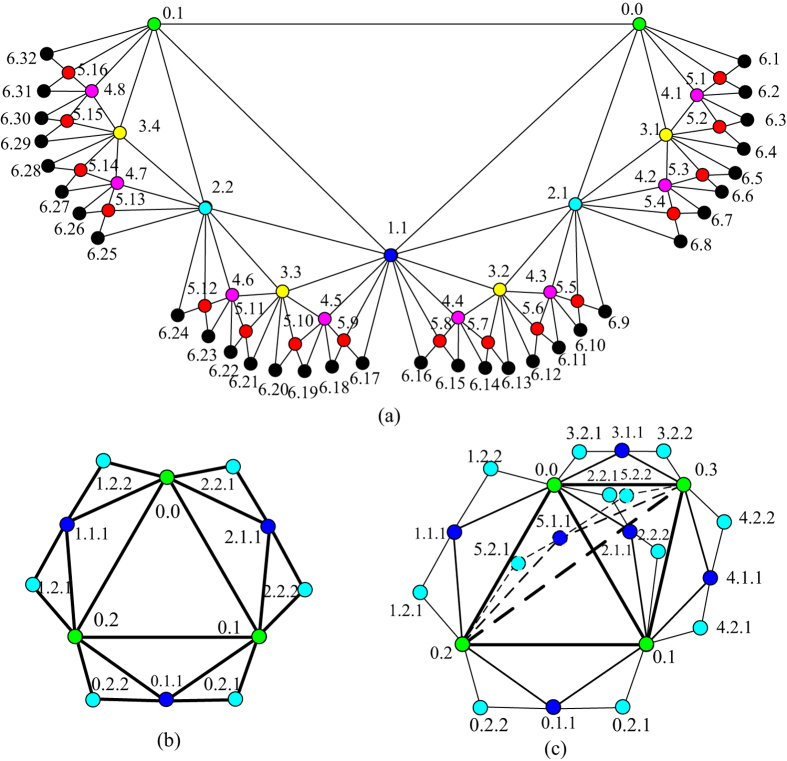
Labels of all vertices in Farey graphs when t = 6, 2 and 2, respectively. (**a**) Farey graphs *F*(6). (**b**) Farey graphs *N*(2). (**c**) Farey graphs *DMG*(2).

The labellings of *F*(6), *N*(2) and *DMG*(2) are illustrated in Fig. 3.

### Routing protocol in *F*(*t*)

To find all the shortest paths between any two vertices in Farey graphs, the relationships between the labels of different vertices should be studied firstly. By the help of vertex labels, these relationships are explored by a quantitatively and precisely manner as follows. For convenience, the vertices labelling with 0.0, 0.1 and 1.1 in *F*(*t*) are marked as *Y*_1_, *Y*_2_ and *X*, respectively. The vertex with label 1.1 is denoted as the hub for it has the highest degree in *F*(*t*). Next, we give several properties about the above labelling (the proof of which will be described in the Method section). Assuming two arbitrary vertices in *F*(*t*) are labelled with *t*_*i*_.*k* and *t*_*j*_.*l*.

**Property 1.** (The family of *t*_*i*_.*k*)

- When *t*_*i*_ ≥ 1, the two children of *t*_*i*_.*k* are (*t*_*i*_ + 1).2*k* and (*t*_*i*_ + 1).(2*k* − 1).
- When *t*_*i*_ ≥ 2 and *k* is odd, the brother of *t*_*i*_.*k* is *t*_*i*_.(*k* + 1), else if *k* is even, the brother is *t*_*i*_.(*k* − 1).
- When *t*_*i*_ ≥ 2, the three vertices, vertex *t*_*i*_.*k* and its parents, shape a triangle. The mother is 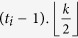, the father is 
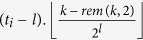
, in which 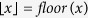 is a function rounding the real number *x* toward negative infinity, *rem*(*k*, 2) keeps the remainder of *k* divided by 2, the integer *l* denotes the sum of one and the number of the continuous zeros from right to left in the binary sequence which is converted by the decimal number *k* − *rem*(*k*, 2). If *t*_*i*_ ≥ *t*_*j*_, the *t*_*i*_ − *t*_*j*_ generations of maternal ancestor of *t*_*i*_.*k* is 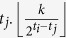.

**Property 2.** (The neighbors of *t*_*i*_.*k*)

- When *t*_*i*_ ≥ 2, the set of neighbor vertices of *t*_*i*_.*k* is 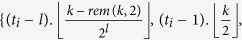 (*t*_*i*_ + *x*).2^*x*−1^(2*k* − 1), (*t*_*i*_ + *x*).[2^*x*−1^(2*k* − 1) + 1]}, where *x* ∈ {1, 2, ..., *t* − *t*_*i*_}.
- The neighbors set of 0.0 is {*t*_*i*_.1}, where *t*_*i*_ ∈ {0, 1, 2, ..., *t*}.
- The set about 0.1 is 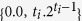, in which *t*_*i*_ ∈ {1, 2, ..., *t*}.
- The set of 1.1 is {0.0, 0.1, (1 + *x*).2^*x*−1^(2*k* − 1), (1 + *x*).[2^*x*−1^(2*k* − 1) + 1]}, *x* ∈ {1, 2, ..., *t* − 1}.

**Property 3.** If any pair of vertices *t*_*i*_.*k* and *t*_*j*_.*l* are located in different subgraphs *F*_1_(*t* − 1) and *F*_2_(*t* − 1) of *F*(*t*) respectively.

• The shortest paths between them pass the hub *X* if

(a).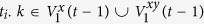 and 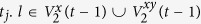,

(b).or 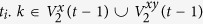 and 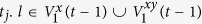,

(c).or 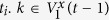 and 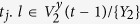,

(d).or 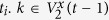 and 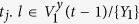.

• The shortest paths go through the two initial vertices *Y*_1_, *Y*_2_ and the edge between them, if

(a).
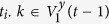
 and 
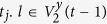
,

(b).or 
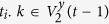
 and 
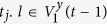
.

• The shortest paths go by *X*, or *Y*_1_ and *Y*_2_, simultaneously, if

(a).
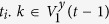
 and 
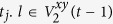
,

(b).or 
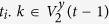
 and 
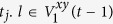
.

**Property 4.** All the shortest paths between any pair of vertices are located in a minimum common subgraph (MCSG) which is denoted as *F*^*mcsg*^(*t*_min_). Moreover, one vertex is positioned in the outermost layer of a subgraph 
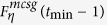
 in *F*^*mcsg*^(*t*_min_), the other vertex is an initial vertex or a vertex seating in the *p* + 1 layer of the other subgraph 
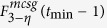
, where *η* = 1, 2.

Here we give the shortest paths routing protocol between any two vertices in Farey graphs *F*(*t*). The algorithm of finding out the shortest paths is unique, as the routing is generated both from each vertex until all vertices in the routing are attained. However, to obtain all the full routing the only information needed are the labels of the source and destination vertices. To find all the shortest paths between any pair of vertices which are labelled with *t*_*i*_.*k* and *t*_*j*_.*l* in Farey graphs, the three main steps are as follows. Firstly, the MCSG *F*^*mcsg*^(*t*_min_) of two target vertices is ascertained. Then, the hub *X* and two initial vertices *Y*_1_ and *Y*_2_ in *F*^*mcsg*^(*t*_min_) are determined whether on the road of the shortest paths or not. Thirdly, the new pairs of vertices are generated which are combined *t*_*i*_.*k* or *t*_*j*_.*l* together with *X* or *Y*_1_ or *Y*_2_. The shortest paths between any pair of vertices are obtained by repeating the three steps till all new vertices pairs are neighbors. The detailed shortest routes in Farey graphs *F*(*t*) are shown as below.

**Routing Algorithm 1.** (SPAF) The shortest path algorithm in *F*(*t*) is shown as below.

Step 1. Given any vertices pair are labelled with *t*_*i*_.*k* and *t*_*j*_.*l* (for the convenience of analysis, assuming *t*_*i*_ ≥ *t*_*j*_).

Step 2. Determine whether the two vertices are neighbors or not.

If *t*_*i*_ − *t*_*j*_ = 1 and 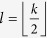, or *t*_*i*_ − *t*_*j*_ = *m* and 
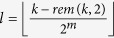
, by property 1, two vertices are the relationship of mother-child or father-child. Insert the two labels to the labels set of the shortest paths (*LSSP*_*m*_(*h*)) and *h* = *h* + 1. Notice that, *h* is the shortest distance between any two vertices, *m* is the number of the shortest paths and *LSSP*_*m*_(0) = ∅. Go to step 6.

Step 3. If the relationship of the two vertices is offspring and maternal ancestor, ascertain the MCSG of two vertices.

If 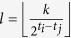, *t*_*j*_.*l* is the *t*_*i*_ − *t*_*j*_ generations maternal ancestor of *t*_*i*_.*k*, then the MCSG is depended on which range the number *k* belongs to.

If 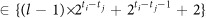, or 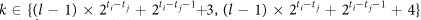, or …, or 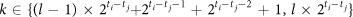, the MCSG is the homomorphic graph from *F*(2) to *F*(*t*_*i*_ − *t*_*j*_), respectively. The vertex *t*_*j*_.*l* is the initial vertex 0.0 and *t*_*i*_.*k* is an outermost layer vertex of MCSG.

Or, if 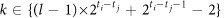, or 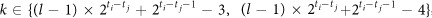, or …, or 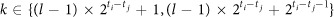, the MCSG is also the homomorphic graph from *F*(2) to *F*(*t*_*i*_ − *t*_*j*_), but *t*_*j*_.*l* is the other initial vertex 0.1 in *F*^*mcsg*^(*t*_min_) and *t*_*i*_.*k* is still an outermost layer vertex in the MCSG.

Go to step 5.

Step 4. Find out the MCSG *F*^*mcsg*^(*t*_min_) when *t*_*j*_.*l* is not the maternal ancestor of *t*_*i*_.*k*.

If 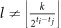, then denote 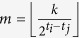, i.e., *t*_*j*_.*m* is the *t*_*i*_ − *t*_*j*_ generations maternal ancestor of *t*_*i*_.*k*. Separate the set 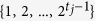 into 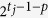 subsets which are with the same order of 2^*p*^ by increasing integer *p* from 0 to *t*_*j*_ − 1, till *m* and *k* belong to a same subset. Then, the MCSG is *F*(*t*_*i*_ − *t*_*j*_ + *p* + 1), and *t*_*i*_.*k* is an outermost layer vertex in subgraphs 
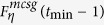
, while *t*_*j*_.*l* is a *p* + 1 layer vertex in the other subgraph 
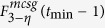
 of *F*^*mcsg*^(*t*_min_). Go to step 5.

Step 5. Confirm hub *X* and two initial vertices *Y*_1_ and *Y*_2_ of *F*^*mcsg*^(*t*_min_) are whether on the shortest paths or not.

Firstly, in order to facilitate the calculation of shortest paths routing on the basis of the labels of vertices in MCSG, the one-to-one correspondence between the labels of two graphs, *F*^*mcsg*^(*t*_min_) and *F*(*t*_min_), is established, for they are isomorphic graphs.

Secondly, all the vertices in *F*(*t*_min_) are divided into six sets by their distances to two initial vertices of 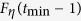: 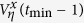, 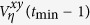 and 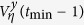, where *η* = 1, 2, and ascertained which sets *t*_*i*_.*k* and *t*_*j*_.*l* belong to.

Then, the three vertices *X*, *Y*_1_ and *Y*_2_ are determined whether on the shortest paths or not by property 3.

If *X* is on the paths, insert the label of *X*, assuming *t*_*p*_.*q*, in the middle of two labels *t*_*i*_.*k* and *t*_*j*_.*l* in the set *LSSP*_*m*_(*h*), and *h* = *h* + 1. Therefore, we can make up two new pairs of labels: *t*_*i*_.*k* and *t*_*p*_.*q*, and *t*_*p*_.*q* and *t*_*j*_.*l*, respectively. Go to step 1.

If the shortest paths pass two initial vertex *Y*_1_ and *Y*_2_, insert the labels of *Y*_1_ and *Y*_2_, *t*_*p*1_.*q*1 and *t*_*p*2_.*q*2, in the middle of *t*_*i*_.*k* and *t*_*j*_.*l* in the set *LSSP*_*m*_(*h*), and *h* = *h* + 2. Two new pairs of labels, *t*_*i*_.*k* and *t*_*p*1_.*q*1, *t*_*p*2_.*q*2 and *t*_*j*_.*l*, are obtained in turn. Go to step 1.

If the shortest paths go through *X* or *Y*_1_ and *Y*_2_, simultaneously, insert *t*_*p*_.*q* into *LSSP*_*m*_(*h*), and *h* = *h* + 1. Then, insert *t*_*p*1_.*q*1 and *t*_*p*2_.*q*2 into *LSSP*_*m*+1_(*h*) and *h* = *h* + 2. The four new pairs of labels is deduced: *t*_*i*_.*k* and *t*_*p*_.*q*, *t*_*p*_.*q* and *t*_*j*_.*l*, *t*_*i*_.*k* and *t*_*p*1_.*q*1, and *t*_*p*2_.*q*2 and *t*_*j*_.*l*, respectively. Go to step 1.

Step 6. Get the shortest paths routing and the distance.

Based on all the sets of *LSSP*_*m*_(*h*), the distance between vertices *t*_*i*_.*k* and *t*_*j*_.*l* is *h*, *m* is the number of the shortest paths, and the shortest routes are exactly traversed every elements in every set of *LSSP*_*m*_(*h*) in order.

**Example 1.** The 10 shortest paths routings from vertices 5.3 to 6.22 in Fig. 3(a) are {5.3, 3.1, 0.0, 0.1, 2.2, 4.6, 6.22}, {5.3, 3.1, 0.0, 1.1, 2.2, 4.6, 6.22}, {5.3, 3.1, 0.0, 1.1, 3.3, 4.6, 6.22}, {5.3, 3.1, 0.0, 1.1, 3.3, 5.11, 6.22}, {5.3, 3.1, 2.1, 1.1, 2.2, 4.6, 6.22}, {5.3, 3.1, 2.1, 1.1, 3.3, 4.6, 6.22}, {5.3, 3.1, 2.1, 1.1, 3.3, 5.11, 6.22}, {5.3, 4.2, 2.1, 1.1, 2.2, 4.6, 6.22}, {5.3, 4.2, 2.1, 1.1, 3.3, 4.6, 6.22} and {5.3, 4.2, 2.1, 1.1, 3.3, 5.11, 6.22}, the distance is 6.

### Routing protocol in *N*(*t*)

On the basis of our efficiently labelling and routing algorithms of Farey graphs *F*(*t*), Farey graphs *N*(*t*) can be labelled and routed similarly. The labelling schematic diagram is shown in Fig. 3(b). The routing algorithm of any two vertices in *N*(*t*) is deduced as below. Supposing any two vertices are labelling with *g*_1_.*t*_*i*_.*k* and *g*_2_.*t*_*j*_.*l*, in which *g*_1_, *g*_2_ ∈ {0, 1, 2}.

**Routing Algorithm 2.** (SPAN) The shortest path algorithm in *N*(*t*) is shown as below.

Step 1. If two vertices are in the same subgraph *F*_*g*_(*t*), i.e., *g*_1_ = *g*_2_ = *g*, the routing of the shortest paths is the same as the algorithm of SRAF above. The shortest paths are obtained by SRAF when the input labels are *t*_*i*_.*k* and *t*_*j*_.*l*.

Step 2. If *g*_1_ ≠ *g*_2_, the two vertices are located in different subgraphs 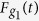 and 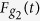. From the recursive construction of *F*(*t*) and *N*(*t*), the two subgraphs above constitute a Farey graphs *F*(*t* + 1). Thus, the routing algorithm is similar as SRAF, but the input labels are (*t*_*i*_ + 1).*k* and 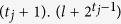 if {*g*_1_, *g*_2_} ∈ {{2, 1}, {1, 0}, {0, 2}}, or, 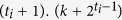 and (*t*_*j*_ + 1).*l* if {*g*_1_, *g*_2_} ∈ {{1, 2}, {0, 1}, {2, 0}}.

### Routing protocol in *DMG*(*t*)

By assuming the label of two arbitrary vertex in *DMG*(*t*) as *g*_1_.*t*_*i*_.*k* and *g*_2_.*t*_*j*_.*l*, in which *g*_1_, *g*_2_ ∈ {0, 1, 2, 3, 4, 5}, the shortest paths routing protocol is similar with SRAN above. The labelling schematic diagram is shown in Fig. 3(c).

**Routing Algorithm 3.** (SPAD) The shortest path algorithm in *DMG*(*t*) is presented below.

Step 1. When *g*_1_ = *g*_2_, the two vertices are in the same subgraph *F*_*g*_(*t*), the shortest paths are obtained just by calling the function of SPAF by inserting *t*_*i*_.*k* and *t*_*j*_.*l* into it.

Step 2. If *g*_1_ ≠ *g*_2_ and {*g*_1_, *g*_2_} ∈ {{0, 1}, {1, 0}, {0, 2}, {2, 0}, {1, 2}, {2, 1}, {0, 4}, {4, 0}, {0, 5}, {5, 0}, {4, 5}, {5, 4}, {2, 3}, {3, 2}, {2, 4}, {4, 2}, {3, 4}, {4, 3}, {1, 3}, {3, 1}, {1, 5}, {5, 1}, {3, 5}, {5, 3}}, two vertices are locating in different subgraphs 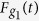 and 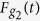, and the two subgraphs share a common initial vertex. This condition is exactly the same as the step 2 of SPAN.

Step 3. If *g*_1_ ≠ *g*_2_ and {*g*_1_, *g*_2_} ∈ {{0, 3}, {3, 0}, {1, 4}, {4, 1}, {2, 5}, {5, 2}}, two vertices are in different subgraphs 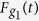 and 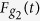 but with no common initial vertex.

Apparently, the four initial vertices of 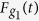 and 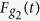 shape a complete graph with four nodes, and the routings in the six conditions above are similar to one another, thus we take {*g*_1_, *g*_2_} = {2, 5} as an example. Divide all the vertices in *F*_2_(*t*) and *F*_5_(*t*) into six sets, 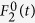, 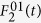, 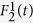, 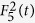, 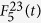 and 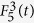, by the distances from them to the two initial vertices which are labelled with 0.0 and 0.1, or, 0.2 and 0.3, respectively.

(1) If 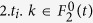: (a) if 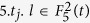, all the shortest paths pass vertices 0.0 and 0.2; (b) else if 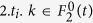 and 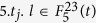, all the paths go through 0.0 and 0.2, or, 0.0 and 0.3; (c) else if 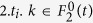 and 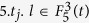, the paths go by 0.0 and 0.3.

(2) If 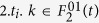: (a) if 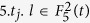, all the shortest paths pass 0.0 and 0.2, or, 0.1 and 0.2; (b) else if 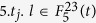, all the paths go through 0.0 and 0.2, 0.1 and 0.2, 0.0 and 0.3, or, 0.1 and 0.3; (c) else if 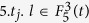, the paths go by 0.0 and 0.3, or, 0.1 and 0.3.

(3) If 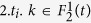: (a) if 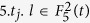, all the shortest paths pass through 0.1 and 0.2; (b) else if 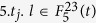, the paths go through 0.1 and 0.2, or, 0.1 and 0.3; (c) else if 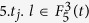, the paths go by 0.1 and 0.3.

(4) Then, all the new pairs of vertices are in the same Farey graphs *F*(*t*); the rest vertices in shortest paths are obtained by SPAF, in which the inserting parameters are just the labels of each new pair of vertices.

## Discussion

We have provided a labelling and routing algorithm for a wide family of Farey graphs, including the model created by edge iterations, evolving networks with geographical attachment preference, general geometric growth model for pseudofractal scale-free web and the networks with multidimensional growth.

The labelling and routing algorithms have several characteristics or advantages. Firstly, our labelling method is simpler than that of refs 12, 15 and 16, because it is easier to deduce the labels of the new vertices which are linked to graphs at step *t*, by our labelling method. Secondly, the routing algorithm of Farey graphs is the first routing algorithm in a deterministic model which has lesser symmetry. General speaking, the lesser symmetry a model has, the harder the properties we can derive. If the symmetry of a deterministic model is defined as the number of similar nodes in the graph, the numbers in Farey graphs and which models of refs 12, 15 and 16 are 2, 3, 4 and 2d, respectively, where d is a positive integer. Apparently, Farey graph has the least symmetry in all these deterministic models. Lastly, it is the first algorithm that *all* the shortest paths are obtained only by their labels in deterministic graphs, while only *one* shortest path is got by similar methods at past literatures.

The time complexity of the routing algorithm in *F*(*t*) is decided by the maximum number of the shortest paths between two vertices, or the steps when the two vertices are adding to graphs. By coincidence, the number is related to the famous Fibonacci numbers. The maximum number is exactly the product of two Fibonacci numbers (*F*_*n*_, in which *F*_*n*_ = *F*_*n*−1_ + *F*_*n*−2_ and *F*_0_ = *F*_1_ = 1). Because the construction mechanism of Farey graphs is recursive, all the vertices on the shortest paths shape rhombuses which are zigzagged adjacent. Moreover, the number reaches maximum only when the two vertices are situated in the different subgroups of Farey graphs: *F*^*x*^(*t*) and *F*^*y*^(*t*). Supposing the labels of these two vertices are *t*_*i*_.*k* and *t*_*j*_.*l*, the maximum number of rhombuses from *t*_*i*_.*k* to 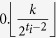 is 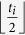, meanwhile, which maximum number from *t*_*j*_.*l* to 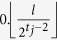 is 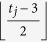. For the rhombuses are staggered adjacent to each other, the number of shortest paths from *t*_*i*_.*k* to 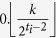 is 
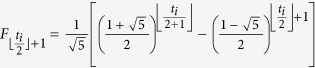
, while the number from *t*_*j*_.*l* to 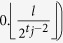 is 
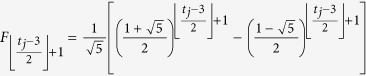
, so that the maximum number is 
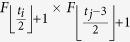
  =   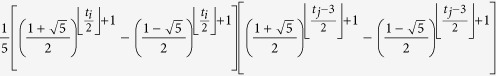. When *t*_*i*_  =  *t*_*j*_  =  *t*, the maximum number in *F*(*t*) is 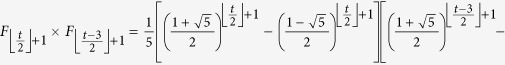

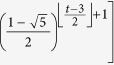
. The number increases almost exponentially. Fortunately, only at most 2*t* + 1 vertices are needed to be ascertained in the routing algorithm of SPAF. For instance, vertices of 0.1, 0.0, 1.1, 2.1, 3.1, 4.2, 5.4 and 6.7 shape three rhombuses, while 0.1, 4.8, 5.16 and 6.31 form one rhombus, so that the number of shortest paths between 6.7 and 6.31 is *F*_4_ × *F*_2_ = 10.

In this algorithm, only five additions and seven multiplications of operations are needed for ascertaining one vertex which is on the shortest paths. As a result, all the shortest paths between any pair vertices can be determined in linear time of *O*(*n*). For arbitrary graphs, the classical algorithms of all-pairs shortest paths problem run in sub-cubic time^13^, while the SPAF for all-pairs in, also only in, Farey graphs runs in sub-quadratic time *O*(*n*^2−*δ*^) at most. Because Farey graphs are composed by special and recursive manner, so that the shortest paths between vertices of inner layers are covered by the shortest paths between vertices which are located on outer layers.

The algorithms can also be extended to the weighted or delayed models, such as weighted scale-free small-world networks^17^, and delayed pseudofractal networks^18^.

## Methods

The routing algorithm of Farey graphs is on the basis of the discovery of several important properties about it; we here proof it in detail.

### The proof of Property 1

The family of a vertex *t*_*i*_.*k* includes a father, a mother, a brother and offspring, the labels for others vertices are very obvious besides the father’s, here we only proof it. If *t*_*i*_ ≥ 2, let Δ*t* denotes the time difference *t*_*i*_ − *t*_*j*_, thus, Δ*t* ∈ {2, ..., *t*_*i*_ − 2}. For *k* = 1 and with any step *t*_*i*_, the fathers are all the vertex 0.0. When *k* is even, the time difference Δ*t* is the sum of one and the number of the continuous zeros from right to left of the binary numbers of *k*, so that the father’s label is 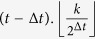; When *k* is odd but excluding one, Δ*t* is the sum of one and the number of the continuous zeros from right to left of the binary numbers of *k* − 1, the father labels with 
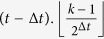
. In summary, when *t*_*i*_ ≥ 2, the father of *t*_*i*_.*k* is marked with 
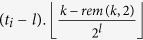
. □

**Remark 2.** The vertex 1.1 has two mothers 0.0 and 0.1, no father and brother. The initial vertices 0.0 and 0.1 have no parents and brother.

### The proof of Property 2

The neighbors of vertex *t*_*i*_.*k* include a father, a mother and 2(*t* − *t*_*i*_) offspring, so that the labels of neighbors can be deduced by Property 1. Here we only proof offspring. For vertices (*t*_*i*_ + *x*).2^*x*−1^(2*k* − 1) and (*t*_*i*_ + *x*).[2^*x*−1^(2*k* − 1) + 1], where *x* ∈ {1, 2, ..., *t* − *t*_*i*_}, the numbers of the continuous zeros from right to left of the binary numbers of 2^*x*−1^(2*k* − 1) − *rem*[2^*x*−1^(2*k* − 1), 2] and 2^*x*−1^(2*k* − 1) + 1 − *rem*[2^*x*−1^(2*k* − 1) + 1, 2] are all *x* − 1 for 2*k* − 1 is odd, so that *l* = *x* in 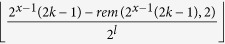 and 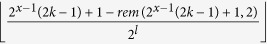, that is to say, (*t*_*i*_ + *x*).2^*x*−1^(2*k* − 1) and (*t*_*i*_ + *x*).[2^*x*−1^(2*k* − 1) + 1] are neighbors of *t*_*i*_.*k*. □

**Remark 3.** The neighbors set of 0.0 is {*t*_*i*_.1}, *t*_*i*_ ∈ {0, 1, 2, ..., *t*}. The set of 0.1 is 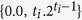, *t*_*i*_ ∈ {1, 2, ..., *t*}. The set of 1.1 is {0.0, 0.1, (1 + *x*).2^*x*−1^(2*k* − 1), (1 + *x*).[2^*x*−1^(2*k* − 1) + 1]}, where *x* ∈ {1, 2, ..., *t* − 1}.

### The proof of Property 3

From the generating algorithm of Farey graph, *F*(*t*) is combined with two subgraphs *F*_1_(*t* − 1) and *F*_2_(*t* − 1), and all the vertices in *F*_*η*_(*t* − 1) (*η* = 1, 2) can be divided into three groups 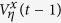, 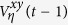 and 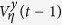 (*η* = 1, 2) by the distance from the vertices in them to the two initial vertices of *V*_*η*_(*t* − 1). The vertices in 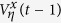 have shorter distance to initial vertex *X* than to *Y*. In contrast, the vertices in 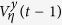 have shorter distance to *Y* than to *X*. If the distances are equal, the vertices are in 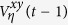. Because *X* and *Y* are neighbors which are linked together, the distances difference is 0 or 1. Therefore, if 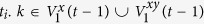 and 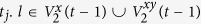, the route between *t*_*i*_.*k* and *t*_*j*_.*l* may go by *X*, or by *Y*_1_ and *Y*_2_, but the distances, if by *X*, are one or two or three shorter than by *Y*_1_ and *Y*_2_, so that the shortest paths in this condition should pass *X*. But in the occasion of 
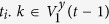
 and 
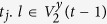
, the paths going by *Y*_1_ and *Y*_2_ are one shorter than by *X*, then the shortest paths go through *Y*_1_ and *Y*_2_. While in case of 
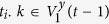
 and 
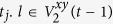
, the paths go by *X*, or *Y*_1_ and *Y*_2_, the distance are equal, so that the shortest paths will go by *X*, or *Y*_1_ and *Y*_2_, simultaneously. Other seven conditions can be proved similarly. □

**Remark 4.** If the sum of the distances from *t*_*i*_.*k* to *X* and from *t*_*j*_.*l* to *X* equals the sum of one and the distances from *t*_*i*_.*k* to *Y*_1_ and from *t*_*j*_.*l* to *Y*_2_, the shortest paths between *t*_*i*_.*k* and *t*_*j*_.*l* go through *X*, or *Y*_1_ and *Y*_2_, at the same time.

### The proof of Property 4

By the construction algorithm of *F*(*t*), all the shortest paths between *t*_*i*_.*k* and *t*_*j*_.*l*, where *t*_*i*_ ≥ *t*_*j*_, are irrelevant to vertices which are added to *F*(*t*) after *t*_*i*_ iterations. Namely, if *t*_*i*_ ≥ *t*_*j*_, all the shortest paths are in the common subgraph *F*(*t*_*i*_). However, the two vertices may located in a common subgraph which is smaller than *F*(*t*_*i*_). The smallest common subgraph, or MCSG *F*^*mcsg*^(*t*_min_), which is contained all the shortest paths between *t*_*i*_.*k* and *t*_*j*_.*l*, is obtained by decreasing *t*_*i*_ till the minimum *t*_min_.

If 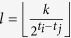, i.e., *t*_*j*_.*l* is the *t*_*i*_ − *t*_*j*_ generations of maternal ancestor of *t*_*i*_.*k*, then, the MCSG is located on both sides of *t*_*j*_.*l* and ascertained precisely by *k*. If *t*_*i*_.*k* is a neighbor of *t*_*j*_.*l*, by Property 2, the MCSG is *F*(0). Else, if 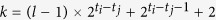, the MCSG is *F*(2); If 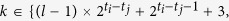

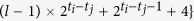
, the MCSG is *F*(3), …, if *k* ∈ {(*l* − 1) × 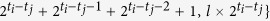, the MCSG is *F*(*t*_*i*_ − *t*_*j*_), where *t*_*j*_.*l* is the initial vertex 0.0 in MCSG. If 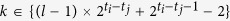, or 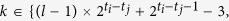 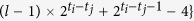 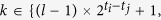 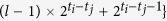, the MCSG is *F*(2), *F*(3), …, *F*(*t*_*i*_ − *t*_*j*_), in turn, and *t*_*j*_.*l* is the other initial vertex 0.1 in *F*^*mcsg*^(*t*_min_). In sum, in this occasion, *t*_*j*_.*l* is an initial vertex of *F*^*mcsg*^(*t*_min_), and *t*_*i*_.*k* is an outermost layer vertex in *F*^*mcsg*^(*t*_min_).

If 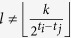, let 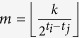, then the position relationship of *l* and *m* determines the MCSG. For 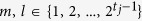, the set 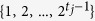 is split evenly into 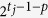 subsets which have the same order of 2^*p*^, till *m* and *k* are located in the same subset by increasing integer *p* from 0 to *t*_*j*_ − 1, then *F*^*mcsg*^(*t*_min_) = *F*(*t*_*i*_ − *t*_*j*_ + *p* + 1), where *t*_*i*_.*k* is an outermost layer vertex and *t*_*j*_.*l* is a *p* + 1 layer vertex which are seated in different subgraph of *F*^*mcsg*^(*t*_min_). □

## Additional Information

**How to cite this article**: Zhai, Y. and Wang, Y. Label-based routing for a family of small-world Farey graphs. *Sci. Rep*. **6**, 25621; doi: 10.1038/srep25621 (2016).

## References

[b1] Comellas F., Ozon J. & Peters J. G. Deterministic small-world communication networks. Inform Process Lett. 76, 83–90 (2000).

[b2] Barabási A. L., Ravasz E. & Vicsek T. Deterministic scale-free networks. Physica A. 299, 559–564 (2001).

[b3] Andrade J. S. Jr, Herrmann H. J., Andrade R. F. S. & Da Silva L. R. Apollonian networks: Simultaneously scale-free, small world, Euclidean, space filling, and with matching graphs. Phys. Rev. Lett. 94, 018702 (2005). 15698147 10.1103/PhysRevLett.94.018702

[b4] Zhang Z. . Mapping Koch curves into scale-free small-world networks. J Phys A-Math Theor. 43, 395101 (2010).

[b5] Zhou T., Wang B. H., Hui P. M. & Chan K. P. Topological properties of integer networks. Physica A. 367, 613–618 (2006).

[b6] Zhang Z. & Comellas F. Farey graphs as models for complex networks. Theor Comput Sci. 412, 865–875 (2011).

[b7] Zhang Z., Wu B. & Lin Y. Counting spanning trees in a small-world Farey graph. Physica A. 391, 3342–3349 (2012).

[b8] Zhang Z., Rong L. & Guo C. A deterministic small-world network created by edge iterations. Physica A. 363, 567–572 (2006).

[b9] Zhang Z., Rong L. & Comellas F. Evolving small-world networks with geographical attachment preference. J Phys A-Math Theor. 39, 3253 (2006).

[b10] Zhang Z., Rong L. & Zhou S. A general geometric growth model for pseudofractal scale-free web. Physica A. 377, 329–339 (2007).

[b11] Peng A. & Zhang L. Deterministic multidimensional growth model for small-world networks. (2011) Available at: http://arxiv.org/ftp/arxiv/papers/1108/1108.5450.pdf (Accessed: 24th November 2015).

[b12] Zhang Z. . Vertex labelling and routing in expanded Apollonian networks. J Phys A-Math Theor. 41, 035004 (2008).

[b13] Chan T. M. More algorithms for all-pairs shortest paths in weighted graphs. SIAM J Comput. 39, 2075–2089(2010).

[b14] Yen J. Y. Finding the K-Shortest Loopless Paths in a Network. Manage Sci. 17 712–716 (1971).

[b15] Comellas F. & Miralles A. Vertex labelling and routing in self-similar outer-planar unclustered graphs modeling complex networks. J Phys A-Math Theor. 42, 425001 (2009).

[b16] Comellas F. & Miralles A. Label-based routing for a family of scale-free, modular, planar and unclustered graphs. J Phys A-Math Theor. 44, 205102 (2011).

[b17] Zhang Y., Zhang Z., Zhou S. & Guan J. Deterministic weighted scale-free small-world networks. Physica A. 389, 3316–3324 (2010).

[b18] Sun W., Wu Y., Chen G. & Wang Q. Deterministically delayed pseudofractal networks. J Stat Mech-Theory E. 10, P10032 (2011).

